# Selective B-cell subset depletion underlies increased infection risk in patients with MM treated with anti-BCMA vs anti-GPRC5D bsAbs^∗^

**DOI:** 10.1182/blood.2025029572

**Published:** 2025-12-19

**Authors:** Tomas Jelinek, David Zihala, Aintzane Zabaleta, Ioannis V. Kostopoulos, Ondrej Soucek, Ondrej Venglar, Cristina Moreno, Despina Fotiou, Eva Radova, Luis Esteban Tamariz-Amador, Foteini Theodorakakou, Ludmila Muronova, Andrea Manubens, Ourania Tsitsilonis, Tereza Popkova, Carmen Gonzalez, Anjana Anilkumar Sithara, Francesco Corrado, Nayda Bidikian, Camila Guerrero, Veronika Kapustova, Daniel Bilek, Patrick R. Hagner, Marta Larrayoz, Jose A. Martinez Climent, Lucie Broskevicova, Jana Mihalyova, Maximilian Merz, Tereza Sevcikova, Irene M. Ghobrial, Jesus San Miguel, Meletios A. Dimopoulos, Paula Rodriguez-Otero, Jakub Radocha, Efstathios Kastritis, Bruno Paiva, Roman Hajek

**Affiliations:** 1 Department of Hematooncology, University Hospital Ostrava, Ostrava, Czech Republic; 2 Department of Hematooncology, Faculty of Medicine, University of Ostrava, Ostrava, Czech Republic; 3 Sylvester Comprehensive Cancer Center, Myeloma Research Institute, University of Miami, Miami, FL; 4 Department of Biology and Ecology, Faculty of Science, University of Ostrava, Ostrava, Czech Republic; 5 Cancer Center Clinica Universidad de Navarra, Centro de Investigación Médica Aplicada Universidad de Navarra, Instituto de Investigación Sanitaria de Navarra, Pamplona, Spain; 6 Department of Biology, School of Sciences, National and Kapodistrian University of Athens, Athens, Greece; 7 Department of Immunology, University Hospital Hradec Kralove and Faculty of Medicine in Hradec Kralove, Charles University, Hradec Kralove, Czech Republic; 8 Plasma Cell Dyscrasia Unit, Department of Clinical Therapeutics, National and Kapodistrian University of Athens, Athens, Greece; 9 Center for Early Detection and Interception, Medical Oncology, Dana-Farber Cancer Institute, Boston, MA; 10 Bristol Myers Squibb, Summit, NJ; 11 Myeloma Service, Memorial Sloan Kettering Cancer Center, New York, NY; 12 Department of Clinical Therapeutics, School of Medicine, National and Kapodistrian University of Athens, Athens, Greece; 13 Department of Medicine, Korea University, Seoul, South Korea; 14 Fourth Department of Internal Medicine-Hematology, University Hospital Hradec Kralove and Faculty of Medicine in Hradec Kralove, Charles University, Hradec Kralove, Czech Republic

## Abstract

Infections remain a challenge during treatment of patients with multiple myeloma (MM) with anti–B-cell maturation antigen (BCMA) and anti–G protein-coupled receptor class C group 5 member D (GPRC5D) bispecific antibodies (bsAbs). However, the mechanism underlying different rates and severity of infections induced by the 2 bsAbs remains poorly understood. Single-cell RNA sequencing of bone marrow (BM) aspirates of 11 patients with MM and 8 healthy donors revealed *BCMA* expression on mature B cells and, surprisingly, in small pre-B cells within B-cell precursors. *GPRC5D* expression was restricted to malignant and less to normal plasma cells (PCs). Next-generation flow cytometry immune profiling showed that anti-BCMA bsAbs severely depleted BM mature B cells, from 4.9% to 0% (*P* < .001), and normal PCs, from 0.17% to <0.0002% (*P* < .001), during treatment of 62 patients with relapsed MM. This was observed throughout therapy. Additional flow cytometry (n = 31) and single-cell RNA sequencing studies (n = 8) demonstrated that, in contrast to anti-GPRC5D, anti-BCMA bsAbs also depleted immature and small pre-B cells. The MIcγ1 mouse model was used as a negative control of *BCMA* expression in all stages of the B-cell lineage, confirming no depletion of any B-cell subset after anti-BCMA treatment. In conclusion, we show that although GPRC5D bsAbs selectively target PCs, anti-BCMA bsAbs target both PCs and B cells from the small pre-B stage onward. Our study provides mechanistic insight into the increased infection risk with anti-BCMA therapy and supports individualized bsAb strategies in MM. Moreover, dual targeting of B cells and PCs may have therapeutic potential in other B-cell malignancies or autoimmune diseases.

## Key Points

• Although GPRC5D is highly expressed on tumor PCs and less on normal PCs, BCMA is detectable throughout the B-cell lineage.
• In contrast to anti-GPRC5D, anti-BCMA bsAbs induce depletion of B-cell precursors, mature B cells, and normal PCs.

## Visual Abstract

**Figure:**
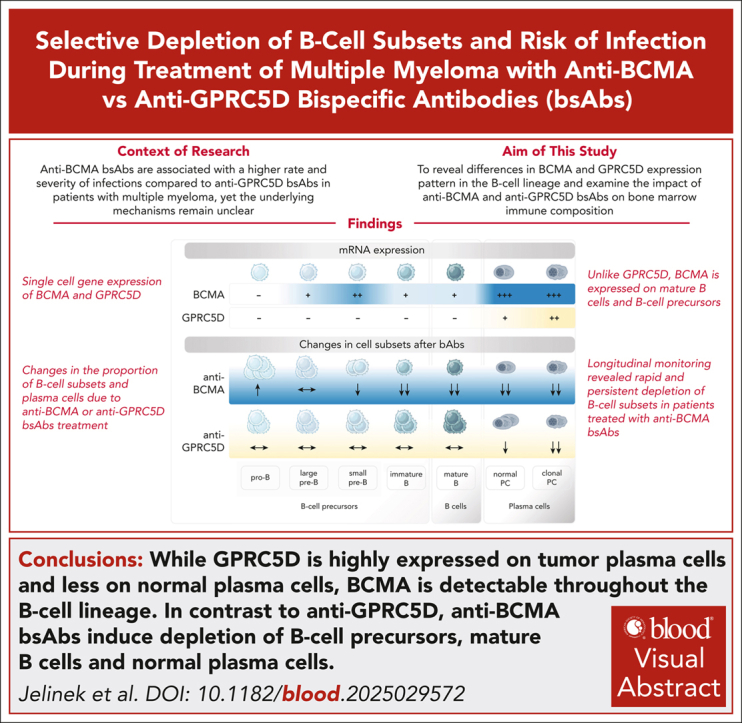

## Teaser

Anti–B-cell maturation antigen (BCMA) bispecific antibodies (bsAbs) are transforming treatment for relapsed multiple myeloma (MM), but infection is a leading cause of morbidity and mortality with these agents in this setting. Jelinek et al help explain this by demonstrating that BCMA is expressed from the early pre-B-cell maturation stage onwards, and consequently anti-BCMA bsAbs deplete B-cell subsets as well as plasma cells, especially mature B cells. In contrast, the expression of G protein–coupled receptor class C group 5 member D (GPRC5D) is highly restricted to malignant and normal plasma cells, and the data from this study suggest that, while still common, infections are less frequent with anti-GPRC5D bsAb therapy.

## Introduction

Bispecific antibodies (bsAbs) represent one of the most significant recent advances in the treatment of relapsed/refractory (R/R) multiple myeloma (MM). These bsAbs simultaneously target CD3 on T cells and either B-cell maturation antigen (BCMA) or G protein–coupled receptor class C group 5 member D (GPRC5D) on plasma cells (PCs). Currently, the anti-BCMA teclistamab, elranatamab, and linvoseltamab as well as the anti-GPRC5D talquetamab are approved therapies for patients with R/R MM.^1^, ^2^, ^3^, ^4^ Their use in combination with other drugs in the R/R and frontline settings is eagerly anticipated, as well as the simultaneous targeting of both antigens to improve efficacy and patient survival.^5^

Infections represent a critical challenge associated with bsAb therapy. Hypogammaglobulinemia, T-cell exhaustion, neutropenia, and lymphopenia represent the most relevant causes of infection. Increasing body of evidence suggests that targeting BCMA portends significantly higher rate and severity of infections than targeting GPRC5D.^6^, ^7^, ^8^, ^9^ In the registrational studies of teclistamab and elranatamab, any-grade infection rate was ∼70% to 80%; grade 3 or 4 infection rates ranged from 40% to 55%, and grade 5 occurred in ∼10% of patients.^2^^,^^3^^,^^10^ The incidence appears to be lower with talquetamab; any-grade infection rates ranges from 59% to 73% of patients depending on dose and schedule, grade 3 or 4 was ∼20%, and 2% of patients died due to infection.^1^^,^^11^^,^^12^ One of the reasons may be on-target, off-tumor toxicity associated with different expression patterns of BCMA and GPRC5D,^13^, ^14^, ^15^, ^16^, ^17^, ^18^, ^19^ but this has not been investigated in sufficient detail.

BsAbs are currently being evaluated in the frontline setting and in patients with smoldering MM, making the risk benefit ratio associated with their use even more critical. Thus, the mechanisms responsible for the development of infections must be well understood. Here, we analyzed the effects of anti-BCMA and anti-GPRC5D bsAbs on the bone marrow (BM) immune composition and uncovered differences between BCMA and GPRC5D expression patterns throughout the B-cell lineage. These findings provide a mechanistic explanation underlying the higher infection rate in patients with MM treated with anti-BCMA bsAbs.

## Methods

### Patients and data collection

This multicenter study included a total of 178 patients with R/R MM treated with anti-BCMA (teclistamab or elranatamab; n = 86) or anti-GPRC5D bsAbs (talquetamab; n = 92) at 4 European academic centers (Athens, Greece; Pamplona, Spain; Hradec Kralove and Ostrava, Czech Republic) between 2017 and 2025 (Table 1). Patients were treated either as monotherapy or in combination with anti-CD38 monoclonal antibodies or immunomodulatory drugs (supplemental Tables 2 and 3, available on the *Blood* website). Clinical data were extracted using retrospective chart review. Infections were evaluated according to Common Terminology Criteria for Adverse Events 5.0. Cytogenetic abnormalities were determined by fluorescence in situ hybridization, and high-risk was defined by the presence of at least 1 of the following: t(4;14), t(14;16), or del(17p). Extramedullary disease was considered if involving soft tissues nonadjacent to bones.^20^ Immunoglobulin levels were measured per institutional guidelines.

**Table 1.** **Brief description of cohorts used in the study**

Description	Method	No. of patients	BCMA (n)	GPRC5D (n)	References
Main multicenter cohort of patients with R/R MM treated with bsAbs (paired baseline and MRD samples)	FCM-NGF	99 (62)	36 (24)	63 (38)	Figure 1; supplemental Tables 1 and 2
Longitudinal BM monitoring (≥3 time points)	FCM-NGF	13	7	6	Figure 2; supplemental Table 3
Longitudinal PB monitoring (baseline, d7, d14, d30, d60, d90, and d180)	Spectral cytometry-OMIP	27	20	7	Figure 2; Table 3
scRNA-seq expression pattern of BCMA and GPRC5D; BM samples of patients with NDMM	scRNA-seq	11	—	—	Figure 3; supplemental Table 3
scRNA-seq expression pattern of BCMA and GPRC5D; BM samples of HDs	scRNA-seq	8	—	—	Figure 3; supplemental Methods
Protein BCMA expression, BM of patients with NDMM	Spectral cytometry; 12 color	24	—	—	Figure 3
Effect of bsAbs on BM B-cell precursors	FCM; 9 color	31	19	12	Figure 4; supplemental Table 3
ScRNA-seq validation on BM CD19^+^ cells	scRNA-seq	8	4	4	Figure 4; supplemental Table 3

FCM, flow cytometry; NGF, next-generation flow cytometry; OMIP, Optimized Multicolor Immunophenotyping Panel.

For longitudinal studies analyzing the BM immune composition, we identified 13 patients with MM with ≥3 BM aspirates performed during follow-up. For peripheral blood (PB) longitudinal monitoring, we selected 27 patients with MM with available samples on a weekly basis after initiation of therapy. For further dissection of the B-cell precursor compartment, another cohort of 31 (flow cytometry) and 8 (single-cell RNA sequencing [scRNA-seq]) patients with MM treated with bsAbs was included (supplemental Table 3). The study was conducted in accordance with the principles of the Declaration of Helsinki, and all patients provided written informed consent before sample collection for correlative studies. All the samples and data were processed following standard operating procedures approved by ethical and scientific committees of the respective institutions.

### Single-cell gene expression of BCMA and GPRC5D

scRNA-seq and B-cell receptor sequencing (BCR-seq) were performed in 11 patients with newly diagnosed MM (NDMM) (University of Navarra, Pamplona, Spain; supplemental Table 3). The B-cell lineage was isolated from BM aspirates using fluorescence-activated cell sorting according to the expression of CD19, CD38, CD45, and CD138. Cells were sorted using BD FACSAria III (BD Biosciences). scRNA-seq and BCR-seq were performed using 10X Genomics Single Cell 5′ Solution v1 and v2 following the manufacturer’s protocol (10X Genomics). Libraries were sequenced in a NextSeq550 (Illumina) and a HiSeqX (Illumina; supplemental Material). External validation of the findings in normal B-cell subsets and normal PCs was performed using a publicly available data set of scRNA-seq data obtained from BM aspirates of 8 healthy donors (HDs; Broad Institute, Boston, MA; supplemental Material).

To validate the findings obtained by flow cytometry, we performed scRNA-seq of BM samples from 8 patients with MM treated with either anti-BCMA (n = 4) or anti-GPRC5D bsAbs (n = 4; University Hospital Ostrava, Ostrava, Czech Republic; supplemental Table 3). B cells and normal PCs were sorted from BM mononuclear cells by fluorescence-activated cell sorting according to the expression of following markers: CD19, CD45, CD38, CD138, and Sytox Blue. Cells were sorted using BD FACSAria III (BD Biosciences). Cell suspensions were processed with the Chromium Next GEM Single Cell 3′ RNA Reagent Kit v3.1, dual indexing (catalog no. 1000269; 10X Genomics), according to the manufacturer’s instructions (14 polymerase chain reaction cycles). Libraries were sequenced by Macrogen Europe (Amsterdam, The Netherlands) on an Illumina platform. Sequencing yielded an estimated average of 2916 cells per patient (range, 609-4609), with a mean of 14 318 reads per cell and a median of 3174 genes per cell across patients. Single-cell data analysis is described in detail in the supplemental Methods.

### Immunophenotyping of tumor and immune cells

The BM tumor and immune compositions were analyzed before the initiation of bsAb-containing therapy (baseline, n = 99) and during treatment when minimal residual disease (MRD) evaluation was indicated (MRD time point, n = 62; all paired with baseline samples). Both baseline and MRD assessments were performed using next-generation flow cytometry according to the EuroFlow guidelines.^21^^,^^22^ The median time from baseline to MRD assessment was 5.2 months (interquartile range, 2.8-10.4). Immune subsets were analyzed using a previously defined strategy^23^ that allowed for the quantification of B-cell precursors, naïve and memory mature B cells, as well as normal and tumor PCs, plus CD19^–^/CD56^–^ T cells and CD19^–^/CD56^+^ natural killer (NK)/NK-T cells (both CD27^+^ and CD27^–^), neutrophils, monocytes, and other subsets (supplemental Material).

Longitudinal monitoring of the B-cell compartment in PB was performed in 27 patients with MM treated with bsAbs (BCMA, n = 20; GPRC5D, n = 7) using a 40-parameter/37-color spectral flow cytometry panel from the Optimized Multicolor Immunophenotyping Panel (OMIP) collection, which was developed and optimized for a 5-laser Cytek Aurora System.^24^ In addition, a 9-color conventional flow cytometry panel was developed to discriminate pro-B, large and small pre-B, and immature B cells within the B-cell precursor compartment,^25^, ^26^, ^27^ which was analyzed in BM aspirates from 31 patients with MM (BCMA, n = 19; GPRC5D, n = 12; supplemental Figures 1-3). Evaluation of surface BCMA expression on BM B-cell subsets, including B-cell precursor stages, mature B cells, and PCs, was performed using 12-color spectrum flow cytometry in patients with NDMM (n = 24; supplemental Figure 4).

### The MIcγ1 mouse model

MIcγ1 mice were generated as previously reported^28^ by crossing B6(Cg)-*Gt(ROSA)26Sortm4(Ikbkb)Rsky*/J mice with constitutively active NF-κB signaling by *IKBKB* expression and green fluorescent protein (GFP) expression, C57BL/6N-Gt(ROSA)26Sortm13(CAG-MYC,-CD2∗)Rsky/J mice with *c-MYC* expression, and cγ1-cre mice. To induce the formation of GFP^+^ transgenic PCs in mice housed under specific-pathogen–free conditions, animals were subjected to T-cell–mediated immunization with sheep red blood cells administered intraperitoneally at age 8 weeks, injected again every 21 days. Mice progressively developed human-like MM in the BM and died of the disease around age 7 to 8 months. To measure *Tnfrsf17* gene expression in the B-cell lineage, scRNA-seq was performed on GFP^–^CD45^+^ immune cells (including B cells) and GFP^+^CD138^+^B220^–^ MM cells from the BM of MIcγ1 mice once MM was developed at age 7 month (n = 3), following reported methods and analytical procedures.^28^

### In vivo therapy trial and mouse analyses

To test the in vivo effect of anti-BCMA therapy in the B-cell lineage, a murine BCMA × CD3 surrogate was administered to MIcγ1 mice (n = 9), whereas a control arm received isotype antibody (n = 8). Before therapy initiation, tumor burden was estimated according to the immunoglobulin gamma fraction (M spikes) in serum determined by electrophoresis. Once clonal M spikes were detected around age 6 months, BCMA × CD3 or isotype at 1 mg/kg were administered by intraperitoneal injection once weekly for 8 weeks. Therapy responses were determined by comparing serum M spikes at day 0 with those at 4 and 8 weeks after treatment initiation in the 2 cohorts. At these time points, sequential measurement of white blood cell and lymphocytes counts were determined in a LaserCell device, as well as circulating B cells by flow cytometry. Additionally, BM aspirates were performed in tibia from MIcγ1 mice 72 hours before treatment and after the last BCMA × CD3 or isotype dose. The percentages of GFP^+^ MM cells and the different B-cell subsets (pro-B, pre-B, and immature and mature B cells) in BM were quantified in the 2 therapy cohorts using flow cytometry (supplemental Figure 5; supplemental Table 4).

### Statistical analysis

The Wilcoxon rank-sum test and Wilcoxon signed-rank test were used to compare continuous variables in unpaired and paired samples, respectively. Fisher exact test (applied if n <5 in any group) and Pearson χ^2^ test were used for categorical variables. When multiple testing correction was applied, significance was defined as a *P* value <.05 after Benjamini-Hochberg adjustment for false discovery rate. All statistical analyses were conducted in R (v4.2.4), primarily using the tidyverse (v2.0.0) for data manipulation and the gtsummary (v1.6.2) package for statistical summary.

## Results

### Higher rate of infections and use of immunoglobulin prophylaxis with anti-BCMA vs anti-GPRC5D bsAbs

In total, 99 patients with R/R MM were treated either with anti-BCMA (n = 36) or anti-GPRC5D bsAb–containing regimens (n = 63). Both groups were well balanced regarding the number of previous lines of therapy (median, 3), age, International Staging System distribution, cytogenetic risk, or presence of extramedullary disease (supplemental Table 1). The frequency of any-grade infection was significantly higher in the BCMA vs GPRC5D group (79% vs 54%; *P* = .019) whereas the incidence of grade 3 or 4 infections was comparable (33% vs 34%; *P* = .935). There was 1 death attributed to severe infection in each group. There was no significant reduction of immunoglobulin G (IgG) levels in patients treated with anti-BCMA bsAbs owing to the more frequent use of immunoglobulin replacement therapy (IgRT) in this vs the GPRC5D group (71% vs 33%; *P* < .001). Overall, our series is representative of the accumulating evidence that anti-BCMA bsAbs are associated with deeper immune suppression.

### BCMA, unlike GPRC5D, is expressed on mature B cells and B-cell precursors

**Figure 1.**
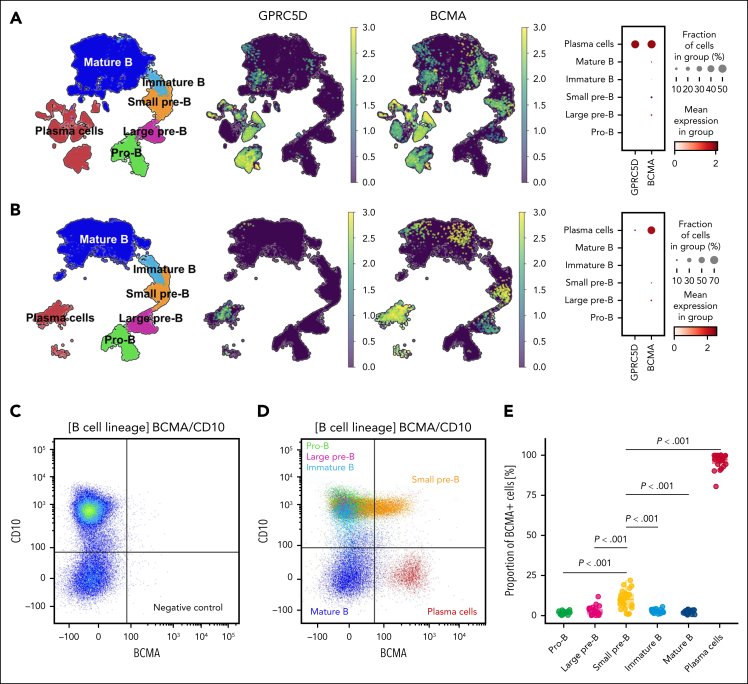
**scRNA-seq analysis of BCMA and GPRC5D expression across mature B cell and B-cell precursor subsets.** (A-B) Uniform manifold approximation and projection visualizations illustrate the overall distribution of analyzed cell types, highlighting expression patterns of GPRC5D and BCMA, with accompanying dot plots representing gene expression levels across distinct cellular subsets in newly diagnosed patients with MM (n = 11; A) and in HDs (n = 8; B). Each dot’s size corresponds to the proportion of cells expressing the gene, and color intensity indicates the average expression level. (C-D) BCMA surface expression analyzed by spectral cytometry in B-cell subsets and PCs using previously defined gating and negative controls. (E) Apart from PCs, BCMA positivity was detected only in a fraction of small pre-B cells, using the phycoerythrin-conjugated antibody clone 19F2.

To elucidate whether the higher rate of infections with anti-BCMA vs anti-GPRC5D bsAbs was a consequence of a distinct pattern of target expression, we analyzed scRNA-seq data obtained from BM aspirates of 11 patients with NDMM. High *BCMA* expression was observed in both clonal PCs from patients with NDMM and normal PCs from HDs (Figure 1). In contrast, *GPRC5D* expression was significantly lower in normal PCs from HDs than clonal PCs from patients with NDMM. Moreover, when we dissected clonal and residual normal PCs in patients with NDMM using BCR-seq, we again observed that *GPRC5D* expression is significantly lower in normal vs clonal PCs, whereas *BCMA* expression was comparable (supplemental Figure 6).

In contrast to *GPRC5D*, which was exclusively expressed in PCs, low levels of *BCMA* messenger RNA were detected in mature B cells as well as in B cell precursors, including immature B cells and small and large pre-B cells, with the highest expression observed in the small pre-B subset (Figure 1A). These observations were validated in HDs (Figure 1B; supplemental Figure 7). To confirm this finding at the protein level, we used spectral flow cytometry in BM samples from 24 patients with NDMM and demonstrated a significantly higher percentage of BCMA^+^ cells and median fluorescence intensity levels within the small pre-B subset than other B-cell subsets (Figure 1C-E; supplemental Figure 8).

### Anti-BCMA bsAbs induce depletion of mature B cells and normal PCs

The unexpected finding of BCMA expression on mature B cells and B-cell precursors led us to investigate the BM immune composition at baseline and MRD assessments throughout treatment with anti–BCMA- and anti–GPRC5D-containing regimens. At baseline, the proportions of neutrophils, monocytes, eosinophils, basophils, T cells (CD19^–^/CD56^–^), NK/NK-T cells (CD19^–^/CD56^+^), mature B cells, and B-cell precursors plus normal and tumor PCs in BM were comparable between the BCMA and GPRC5D groups (data not shown).

**Figure 2.**
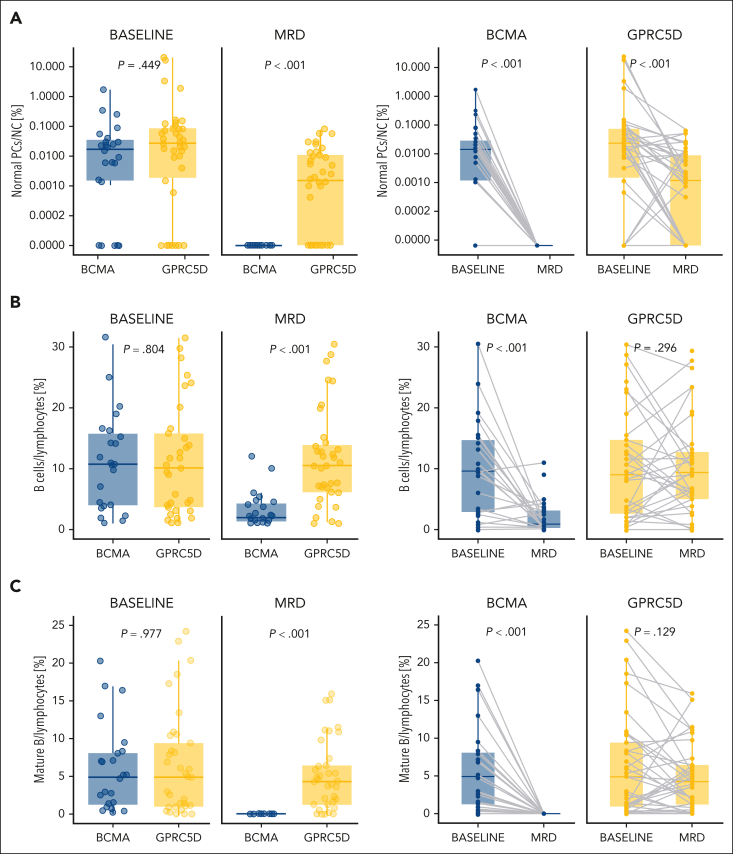
**Comparison of the deregulated immune cell subsets in patients with R/R MM treated with anti-BCMA vs anti-GPRC5D bsAbs using next-generation flow cytometry before the initiation of the therapy (baseline) and during the therapy when MRD assessment was indicated (MRD).** Upper box plots show comparisons between anti-BCMA and anti-GPRC5D samples for patients with both time points available (n = 62), whereas lower box plots show paired comparisons between baseline and MRD for BCMA (n = 24) and GPRC5D (n = 38) separately for total B cells expressed from total lymphocytes (A); mature B cells expressed from total lymphocytes (B); and normal PCs expressed from total BM nucleated cells (C).

The percentage of normal PCs decreased during treatment in both groups. However, in patients treated with anti-BCMA bsAbs, there was complete depletion (from 0.017% to <0.0002%; *P* < .001), whereas in the GPRC5D group, the reduction was less profound (from 0.027% to 0.002%; *P* < .001; Figure 2A). Interestingly, there was a reduction in total B cells and mature B cells (including naïve and memory) during treatment, but such a reduction was significant only in patients treated with anti-BCMA bsAbs (Figure 2B-C). Although the median percentage of total B cells within the lymphoid compartment was similar in both groups at baseline (9.6% vs 9.0%), there was a significant drop during treatment in the BCMA vs the GPRC5D group (0.9% vs 9.7%; *P* < .001; Figure 2B). This drop was predominantly caused by the depletion of mature B cells in patients treated with anti-BCMA bsAbs (from 4.9% at baseline to 0% during treatment; *P* < .001), whereas in the GPRC5D group, the percentage of mature B cells remained stable (from 4.9% to 4.3%; *P* = .129; Figure 2C). There was a borderline significant difference toward greater depletion of B-cell precursors during treatment in the BCMA vs GPRC5D groups (*P* = .087; supplemental Figure 9). Of note, there were no differences in the distribution of any other lymphoid and myeloid subsets.

A significant association was observed between decreased number of mature B cells and an increased rate of infections in patients treated with anti-BCMA or anti-GPRC5D monotherapy (*P* = .048; supplemental Figure 10A). The association was even more pronounced when considering only memory B cells (*P* = .026; supplemental Figure 10B).

### Anti-BCMA bsAbs affect the composition of the B-cell precursor compartment

**Figure 3.**
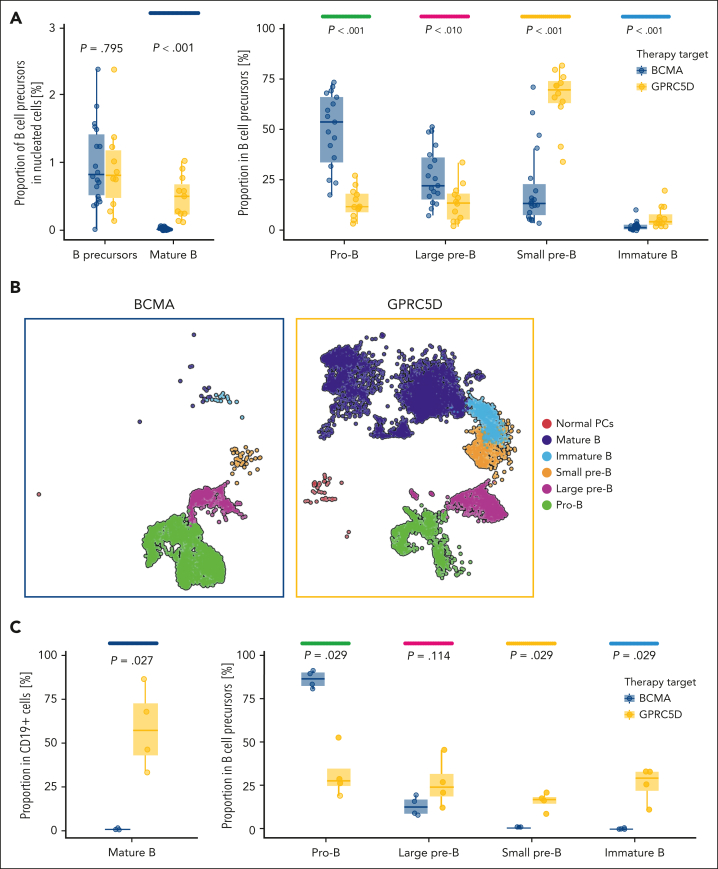
**Comparison of the effects of bsAbs targeting BCMA and GPRC5D on the B-cell and PC compartments.** (A) Proportions of mature B cells, total B-cell precursors, and individual precursor subsets (pro-B, large pre-B, small pre-B, and immature B cells) were analyzed in 31 BM samples (BCMA, n = 19; GPRC5D, n = 12) after therapy using conventional flow cytometry. (B-C) scRNA-seq of CD19^+^ sorted cells from BM of 8 patients (BCMA, n = 4; GPC5D, n = 4) after therapy, shown as UMAP (B) and box plots (C), revealing major differences in precursor subsets between the 2 groups.

After demonstrating that BCMA is expressed in B-cell precursors, we used a flow cytometry panel specifically designed to further dissect the effects of BCMA- and GPRC5D-targeted bsAbs on the various maturation stages present within the B-cell precursor compartment. In total, we analyzed BM samples from 31 patients with MM treated with either anti-BCMA (n = 19) or anti-GPRC5D bsAbs (n = 12). Interestingly, there were significant differences in specific maturational subsets within the B-cell precursor compartment. In the BCMA group, the 2 most mature B-cell precursor subsets (small pre-B and immature B cells) were significantly reduced. In contrast, less mature pro-B cell subsets were significantly increased, whereas the total proportion of B-cell precursors among nucleated cells did not differ between treatment groups (*P* = .8; Figure 3A), suggesting an accumulation of pro-B cells.

To substantiate these findings, we performed scRNA-seq of CD19^+^ fluorescence-activated cell sorted (FACS) cells from BM samples of 8 patients with MM treated with either anti-BCMA (n = 4) or anti-GPRC5D bsAbs (n = 4; Figure 3B). All patients were in complete remission and underwent BM aspiration for MRD assessment. Consistent with the observations of flow cytometry immunophenotyping, depletion of mature B cells and normal PCs was more profound in the BCMA group than the GPRC5D group (Figure 3B-C). Within the B-cell precursor compartment, we again observed significant depletion of immature B cells and small pre-B cells, whereas the pro-B cell subset was significantly enriched.

### Longitudinal monitoring revealed rapid and persistent depletion of B-cell subsets in patients treated with anti-BCMA bsAbs

**Figure 4.**
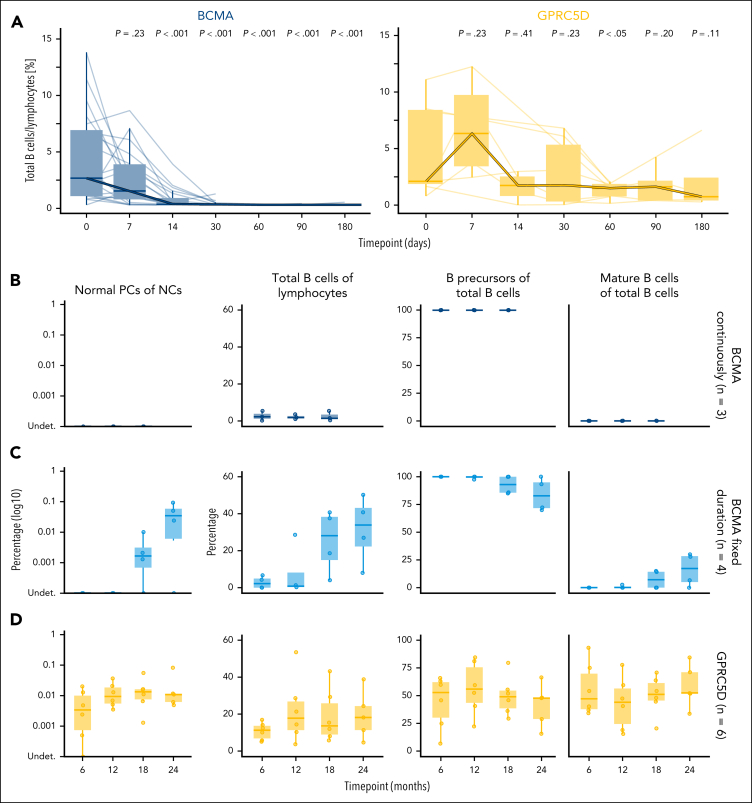
**Longitudinal monitoring of the effects of bsAbs targeting BCMA and GPRC5D in PB and BM.** (A) Total B cells were assessed longitudinally by spectral cytometry panel (OMIP-117) in 161 PB samples (BCMA, n = 122; GPRC5D, n = 39) from patients receiving anti-BCMA (n = 20) or anti-GPRC5D (n = 7), measured at baseline and on days 7, 14, 30, 60, 90, and 180. (B-D) Proportions of normal PCs, total B cells, B-cell precursors, and mature B cells were assessed by NGF in BM MRD samples collected at 6, 12, 18, and 24 months after achieving complete remission in patients who received anti-BCMA bsAbs continuously (B) or with a fixed duration of 12 months (C), as well as patients treated with the anti-GPRC5D bsAb talquetamab (D).

We aimed to determine how quickly mature B cells are depleted in the PB of patients treated with bsAbs. To capture the kinetics of PB B cells, we applied high-dimensional spectral cytometry to 161 samples from 27 patients with MM (BCMA, n = 20; GPRC5D, n = 7) collected at the following time points: baseline (0), day 7, day 14, day 30, day 60, day 90, and day 180. In patients treated with anti-BCMA bsAb, we observed a significant decrease of mature B cells by day 14 (*P* < .001), with their complete disappearance by day 30 and ongoing depletion until day 180 (Figure 4A). Naïve and memory mature B cells followed the same kinetics pattern (supplemental Figure 11). In contrast, in patients treated with anti-GPRC5D bsAb, mature B cells remained stable throughout the monitoring period (Figure 4A).

To investigate the long-term effects of bsAbs on the BM B-cell and PC compartments, we analyzed patients with MM with at least 3 BM aspirates obtained for MRD assessment during follow-up. First, we identified patients treated continuously with teclistamab (n = 3) and observed a persistent and virtually complete depletion of normal PCs and mature B cells throughout therapy (Figure 4B). Next, we evaluated patients treated with teclistamab for a fixed duration of 12 months (n = 4). In these patients, we again detected sustained depletion of mature B cells and normal PCs during treatment; however, after discontinuation of teclistamab (from 12 months onward), both mature B cells and normal PCs reappeared (Figure 4C). In contrast, in patients with MM treated with talquetamab (n = 6), the proportion of these subsets remained stable throughout the entire course of therapy (Figure 4D).

### Absence of BCMA messenger RNA expression predicts no depletion with anti-BCMA bsAb

**Figure 5.**
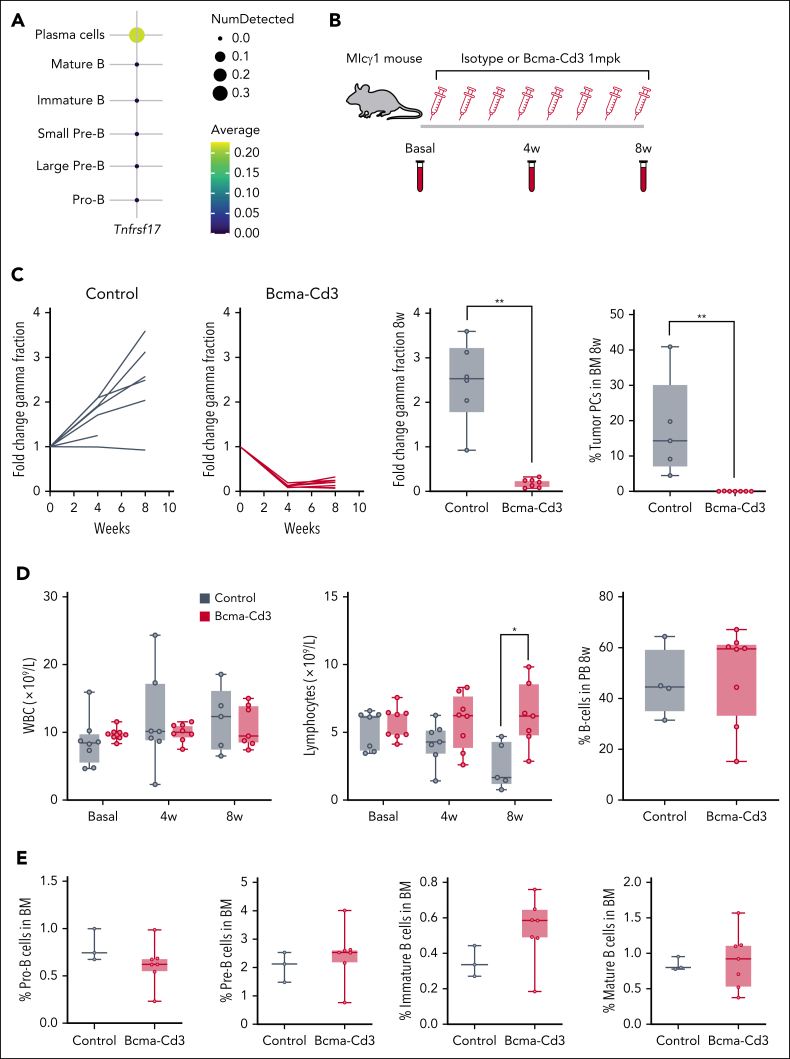
**Preclinical therapy trial testing BCMA × CD3 therapy in MIcγ1 mice.** (A) Levels of expression of BCMA gene across B-cell precursors and mature B-cell subpopulations in untreated MIcγ1 mice, according to scRNA-seq data (see “Methods”). (B) Schematic representation of the in vivo trial in MIcγ1 mice. Treatment with a murine BCMA × CD3 surrogate bsAb (n = 9) or an isotype antibody (n = 8) was started when signs of MM were detected and continued for 8 weeks. (C) Monitoring of treatment responses according to quantification of immunoglobulin levels in serum before treatment and at 4 and 8 weeks after treatment initiation are shown. Additionally, BM aspirates were conducted in tibia from MIcγ1 mice treated with BCMA × CD3 or isotype 72 hours after the last BCMA × CD3 dose, which showed major reduction in the number of MM cells with respect to isotype-treated mice. (D) Determination of white blood cell (WBC) and lymphocyte counts in PB of mice treated with BCMA × CD3 or isotype before treatment and at weeks 4 and 8 after treatment initiation. On the right, measurement of circulating B cells in mice at week 8 after treatment initiation. (E) Quantification of B-cell precursors and mature B-cell subpopulations in the BM of MIcγ1 mice in the 2 treatment arms by multiparametric flow cytometry at week 8 after treatment initiation.

To corroborate the accuracy of scRNA-seq data in terms of detectable *BCMA* expression and functional effect of anti-BCMA treatment, we used the *MIcγ1* mouse model that progressively develops human-like MM at age 7 to 8 weeks. We performed scRNA-seq in BM samples from untreated MIcγ1 mice once MM was developed at 7 months. scRNA-seq analysis showed that, in contrast to humans, *Tnfrsf17* expression was detected only in GFP^+^ MM cells but not in any mature B cells or B precursor stages (Figure 5A).

Next, a murine BCMA × CD3 surrogate was administered to MIcγ1 mice during 8 consecutive weeks; as controls, a second cohort of MIcγ1 mice received an isotype antibody (Figure 5B). BCMA × CD3 therapy induced therapeutic responses, as shown by the decline of serum immunoglobulins that led to major reduction of MM cells in the BM at the end of treatment compared to control mice, which reflect similarities with therapeutic responses in patients (Figure 5C). In contrast, BCMA × CD3 did not affect the number of white blood cells or lymphocytes nor did it alter the percentages of circulating B cells (Figure 5D). Additional flow cytometry analyses showed no statistically significant differences in the percentages of pro-B, pre-B, immature, or mature B cells in the BM between BCMA × CD3-treated and control mice (Figure 5E). These experiments in the MIcγ1 model showed that in the absence of *Bcma* expression on precursor/mature B cells, there is no depletion of these subsets with BCMA-targeting therapy.

## Discussion

**Figure 6.**
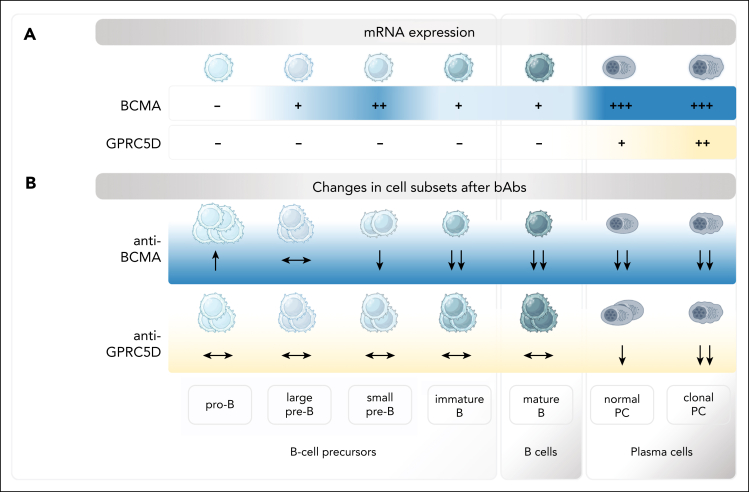
**Schematic overview of BCMA and GPRC5D expression across the B-cell lineage and bsAb treatment-induced subset changes.** (A) Illustrative figure of RNA expression levels of BCMA and GPRC5D across the B-cell lineage subsets, from the most immature Pro-B cells to normal and clonal PCs. (B) Changes in the proportion of particular B-cell subsets and PCs due to anti-BCMA or anti-GPRC5D bsAb treatment.

With an increasing number of highly effective treatment options for patients with NDMM and R/R MM, a better understanding of how adverse events originate is paramount for their prevention toward improved quality of life and outcomes. Here, we provide a mechanistic explanation behind a higher rate of infections observed in patients treated with anti-BCMA than anti-GPRC5D bsAbs. Our data suggest that, unlike GPRC5D, BCMA is expressed on the surface of small pre-B cells, which leads to the elimination of this subset and all more mature B-cell stages. Moreover, normal PCs express significantly less GPRC5D compared to tumor PCs, which leads to their persistence during anti-GPRC5D bsAb treatment (Figure 6).

There are contradictory reports about BCMA expression on precursor, naïve, and memory B cells using various methods, although most reports suggest no BCMA expression on precursor and mature B cells.^14^^,^^19^^,^^29^, ^30^, ^31^ Conversely, Frerichs et al reported BCMA staining using flow cytometry on a subset of CD10^+^CD19^+^CD24^+^CD38^+^ B-cell progenitors and mature B cells, which correlated with the depletion of PB mature B cells in patients with R/R MM treated with teclistamab in the MajesTEC-1 trial.^32^ Our data confirm and provide compelling evidence that BCMA expression occurs early during B-cell development. Despite its limited expression, anti-BCMA bsAbs are able to effectively target all B-cell and PC subsets from the small pre-B stage onward, as demonstrated by both flow cytometry and scRNA-seq. Importantly, we used a mouse model as a negative control, in which no expression of *Bcma* in precursor and mature B cells resulted in null effect after anti-BCMA bsAbs.

We also demonstrated that, unlike BCMA, GPRC5D is significantly less expressed on normal PCs compared to clonal PCs, leading to their relative sparing during anti-GPRC5D treatment, as previously suggested by in vitro observations by Verkleij et al and a recent scRNA-seq study by Da Vià et al.^17^^,^^33^ Using longitudinal samples, we showed that depletion of mature B cells and normal PCs after anti-BCMA bsAbs is rapid and persistent throughout treatment, and only its cessation allowed for the regeneration of the B-cell compartment. In contrast, in patients treated with talquetamab, the distribution of these subsets remained stable throughout treatment. These data suggest continuous ongoing suppression of humoral immunity while patients remain on anti-BCMA bsAb therapy.

Our study confirmed that BCMA-targeting bsAbs are associated with higher incidence of any-grade infections. The number of grade 3 or 4 infections was comparable between groups, likely due to the combination of anti-GPRC5D bsAb with either immunomodulatory drugs or anti-CD38 monoclonal antibodies, which could result in increased infection rate. Moreover, the use of IgRT was significantly more frequent in the BCMA group (71% vs 33%; *P* < .001), potentially leading to lower incidence of grade 3 or 4 infections than registrational trials.^1^^,^^2^^,^^8^^,^^10^^,^^11^ These results are timely because the management of patients with MM treated with bsAbs, along with supportive care for infection complications, is being widely discussed, with many recently published recommendations.^34^, ^35^, ^36^ Infections remain the most common cause of nonrelapse mortality, and importantly, the cumulative risk of infections does not plateau with continued time on bsAb therapy.^7^^,^^37^^,^^38^ IgRT, which includes IV and subcutaneous immunoglobulins, may lower these risks. There are in principle 2 strategies: (1) preemptive IgRT (initiated only when IgG levels drops below certain threshold, usually 400 mg/dL) or (2) primary IgRT (initiated regardless of IgG levels), with the latter approach perceived as safer and simpler.^32^^,^^38^^,^^39^ Nevertheless, until now, no recommendation takes into account the targeted antigen. Our data provide a mechanistic foundation for considering a more individualized approach to antiinfective measures in bsAb-treated patients, especially in situations such as IgRT shortage or financial restrains. More importantly, strategies such as fixed-duration therapy, less frequent dosing, or treatment-free intervals may facilitate the recovery of precursor and mature B cells and mitigate the risk of infections.^40^, ^41^, ^42^, ^43^ Our findings may help toward optimized laboratory monitoring of on-target, off-tumor toxicity with routinely used flow cytometry, which may become an important tool for individualized decision-making in the care of patients with MM.

We acknowledge the limitations of our study, such as the retrospective nature of the collected clinical data and the reduced number of patients in certain cohorts. Another important limitation is the use of a murine model, which does not fully reproduce the human physiological context because of different BCMA expression and the use of sheep red blood cells to induce PC proliferation, which poorly reflects the inflammatory and immunological context seen in human MM. Accordingly, the outputs of the murine model have limited translational relevance. In fact, the murine experiments were intended as exploratory and supportive studies to evaluate the sensitivity of scRNA-seq for detecting BCMA expression, rather than as direct validation of findings in humans. Our results indicate that even minimal BCMA expression detected by scRNA-seq can be sufficient to induce B-cell depletion in patients treated with bsAbs, whereas in mice, in which such expression is absent, no depletion is observed. From a clinical perspective, providing evidence that anti-BCMA bsAbs effectively target not only the PC compartment but also B-cell subsets from the small pre-B stage onward may have several implications. It not only explains the higher infection risk associated with anti-BCMA bsAbs but also provides a rationale for individualized IgRT and may guide the development of optimized anti-BCMA bsAb treatment schedules that allow for B-cell regeneration and less toxicity. Moreover, our data may serve as a foundation for the use of BCMA-targeted bsAbs in other B-cell malignancies or even in nonmalignant conditions such as autoimmune diseases, in which targeting both B cells and PCs is therapeutically advantageous.^44^^,^^45^

Conflict-of-interest disclosure: T.J. reports research funding from Janssen and Sanofi, honoraria and consultancy/advisory role for Bristol Myers Squibb (BMS), GSK, Janssen, Pfizer, and Sanofi. J.A.M.C. reports grants from AstraZeneca, BMS, Janssen, Palleon Pharmaceuticals, K36 Therapeutics, Regeneron, Roche, and Priothera Pharmaceuticals; personal fees, research support, and other support from MIMO Biosciences; and a patent for genetically engineered animal models for multiple myeloma (EP23750608.4-PT/EP2023/071025) licensed to MIMO Biosciences. B.P. reports consultancy fees from BMS-Celgene, GSK, Janssen, Roche, Sanofi, and Takeda; research funding from AstraZeneca, BeiGene, BMS, GSK, Roche, and Sanofi; and honoraria from Adaptive, Amgen, Becton/Dickinson Biosciences, BMS-Celgene, GSK, Janssen, Roche, and Sanofi. The remaining authors declare no competing financial interests. M.A.D. reports honoraria from participation in advisory boards and satellite symposia from Amgen, Sanofi, Regeneron, Menarini, Takeda, GSK, BMS, Janssen, Beigene, Swixx, and AstraZeneca. R.H. reports grants or contracts from Amgen, BMS, Celgene, Janssen, Novartis, and Takeda; consulting fees from AbbVie, Amgen, BMS, Celgene, Janssen, Novartis, PharmaMar, and Takeda; honoraria for lectures, presentations, and speaker bureaus from Amgen, BMS, Celgene, Janssen, PharmaMar, and Takeda; support for attending meetings and/or travel from Amgen, Celgene, Janssen, and Takeda; and participation on a data safety monitoring board or advisory board for Amgen, BMS, GSK, Janssen, Oncopeptides, Sanofi, and Takeda. The remaining authors declare no competing financial interests.

## Supplementary Material

Supplemental Methods, Figures, and References
