# Supplementary material for: Selective B-cell subset depletion underlies increased infection risk in patients with MM treated with anti-BCMA vs anti-GPRC5D bsAbs
Source: Blood. 2025 Dec 19;147(10):1070–82. doi: 10.1182/blood.2025029572 (PMC13014095; doi:10.1182/blood.2025029572)
Supplement: Supplemental Methods, Figures, and References [file BLOOD_BLD-2025-029572-mmc1.pdf]

Supplementary materials

Selective depletion of B-cell subsets underlies increased risk of infection in MM patients treated with anti-BCMA vs -GPRC5D bsAbs

Running title: Expression and targeting of BCMA and GPRC5D in MM

Authors

Tomas Jelinek[1,2,3,†,*], David Zihala[1,2,4†], Aintzane Zabaleta[5], Ioannis V. Kostopoulos[6], Ondrej Soucek[7], Ondrej Venglar[1,2], Cristina Moreno[5], Despina Fotiou[8], Eva Radova[1,2], Luis Esteban Tamariz-Amador[5], Foteini Theodorakakou[8], Ludmila Muronova[1,2], Andrea Manubens[5], Ourania Tsitsilonis[6], Tereza Popkova[1,2], Carmen Gonzalez[5], Anjana Anilkumar Sithara[1,2,4], Francesco Corrado[9], Nayda Bidikian[9], Camila Guerrero[5], Veronika Kapustova[1,2], Daniel Bilek[1,2,4], Patrick R. Hagner[10], Marta Larrayoz[5], Jose A. Martinez Climent[5], Lucie Broskevicova[1,2], Jana Mihalyova[1,2], Maximilian Merz[11], Tereza Sevcikova[1,2,4], Irene M Ghobrial[9], Jesus San Miguel[5], Meletios A. Dimopoulos[8], Paula Rodriguez-Otero[5], Jakub Radocha[12], Efstathios Kastritis[8], Bruno Paiva[5,†], Roman Hajek[1,2,†]

† Authors contributed equally

* Correspondence: tomas.jelinek@fno.cz

Affiliations

[1] Department of Hematooncology, University Hospital Ostrava, Ostrava, Czech Republic

[2] Department of Hematooncology, Faculty of Medicine, University of Ostrava, Ostrava, Czech Republic

[3] Sylvester Comprehensive Cancer Center, Myeloma Research Institute, University of Miami, Miami, Florida, USA

[4] Department of Biology and Ecology, Faculty of Science, University of Ostrava, Ostrava, Czech Republic

[5] Cancer Center Clinica Universidad de Navarra (CCUN), CIMA Universidad de Navarra, Instituto de Investigación Sanitaria de Navarra (IDISNA), CIBER-ONC numbers CB16/12/00369, CB16/12/00489, Pamplona, Spain.

[6] Department of Biology, School of Sciences, National and Kapodistrian University of Athens, Athens, Greece

[7] Department of Immunology, University Hospital Hradec Kralove and Faculty of Medicine in Hradec Kralove, Charles University, Hradec Kralove, Czech Republic

[8] Department of Clinical Therapeutics, Plasma cell dyscrasia Unit, National and Kapodistrian University of Athens, Athens, Greece

[9] Center for early detection and interception, Medical Oncology, Dana-Farber Cancer Institute, Boston, MA

[10] Bristol Myers Squibb, Summit, NJ, USA

[11] Myeloma Service, Memorial Sloan Kettering Cancer Center, New York, USA

[12] Fourth Department of Internal Medicine-Hematology, University Hospital Hradec Kralove and Faculty of Medicine in Hradec Kralove, Charles University, Hradec Kralove, Czech Republic

Table of Contents

Supplementary materials *1*

Table of Contents 2

Supplementary Methods *3*

Patients in the multicenter cohort 3

Flow cytometry 3

Single-cell RNA sequencing of NDMM patients and HD 5

Analysis of single cell RNA sequencing data 6

Single-cell RNA comparison of clonal and normal PCs 6

Supplementary Figures *8*

Supplementary References 14

Supplementary Methods

Patients in the multicenter cohort

All patients included in this multi-center study (N=99) were relapsed/refractory multiple myeloma (RRMM) patients with at least one previous line of therapy treated with one of the approved bispecific antibodies (bsAbs) either targeting BCMA (teclistamab, elranatamab) or GPRC5D (talquetamab). No patient received any bsAbs prior to enrollment to the study. Patients were treated as standard of care or within particular clinical trial using the approved/recommended dosing schedule. In total four European academic centers participated in this study: National and Kapodistrian University of Athens, Greece; University of Navarra, Pamplona, Spain; Charles University, Hradec Kralove, Czech Republic and University of Ostrava, Czech Republic; Suppl. Table 1.

Flow cytometry

Fresh bone marrow (BM) aspirates or peripheral blood (PB) samples were collected in EDTA anticoagulated tubes and processed within 24 hours from harvest.

BM immune profiling was performed using the EuroFlow 8-colour conventional flow cytometry diagnostic plasma cell dyscrasia (PCD) panel, in accordance with the EuroFlow standard operating procedures (SOP) (Flores-Montero et al., 2017). Aspirates were assessed either at baseline prior to the initiation of bsAb-containing therapy (N=70), with 1,000,000 target events acquired, or during treatment at the time of minimal residual disease (MRD) assessment (N=40; all paired samples), using next-generation flow (NGF) SOP, with 10,000,000 events acquired (limit of detection 0.0002%). PCD panel composition; tube 1: CD138-BV421, CD27-BV510, CD38-FITC, CD56-PE, CD45-PerCPCy5.5, CD19-PECy7, CD117-APC, CD81-APCH7; and tube 2: CD138-BV421, CD27-BV510, CD38-FITC, CD56-PE, CD45-PerCPCy5.5, CD19-PECy7, cyKAPPA-APC, cyLAMBDA-APCH7. The target number of acquired events was 1,000,000 for baselines and 10,000,000 for MRD. Data acquisition was conducted on a FACSCanto II flow cytometer (BD Biosciences, San Jose, CA, USA) using the FACSDiva 6.1 software (BD Biosciences). Instrument performance was monitored daily using the Cytometer Tracking and Setup Beads (CST; BD) and Rainbow 8-peak beads (SPHERO™ Rainbow Calibration Particles, Spherotech, Lake Forest, IL, USA). Data were analyzed by experienced operators using the Infinicyt software version 2.0 (Cytognos). The PCD panel allows for the accurate discrimination of normal and malignant PCs (normal phenotype: CD45+ CD138+ CD38+ CD19+ CD56- CD117- CD27+ CD81+ Kappa+ and Lambda+; malignant phenotype: CD45+/- CD138+/high/low CD38+/low/- CD19+/- CD56+/- CD117+/- CD81+/low CD27+/low Kappa+ or Lambda+). Besides that, the same panel allows a basic immune profiling

including identification and enumeration of following immune subpopulations used in our study: lymphocytes (CD45+ SSClow), B cell precursors (CD45low CD19+ CD38high), mature B cells (CD45+ CD19+), naïve mature B cells (CD45+ CD19+ CD27-), memory mature B cells (CD45+ CD19+ CD27+), NK/NK-T cells (CD45+ SSClow CD56+ CD19-) divided to CD27+ and CD27-, remaining T cells (CD45+ SSClow CD56- CD19-) divided to CD27+ and CD27-, hematopoietic progenitors (CD45low CD117+ CD38+/-), erythroblasts (CD45- SSClow CD38-), mast cells (CD45low CD117high), eosinophils (CD45high SSChigh CD81high), basophils (CD45high CD38low CD81-), monocytes (CD45+ CD38+ CD81+), neutrophils (CD45low SSChigh CD81-).

For the detailed characterization of B cell precursor compartment (Supp. Figure 1), a conventional 9-colour flow cytometry panel was developed to discriminate pro-B, large pre-B, small pre-B, and immature B cells (Orfao *et al*, 2019) and was implemented in 31 patients (BCMA: N=19, GPRC5D: N=12). Samples were processed with standard flow cytometry praxis and stained with following fluorophore-conjugated monoclonal antibodies: CD10 / ECD (ALB1, Beckman Coulter, Brea, CA, USA); CD34 / PerCP-Cy5.5 (8G12, BD Biosciences); CD19 / PE-Cy7 (J3-119, Beckman Coulter); CD22 / APC (S-HCL-1, BD Biosciences); CD38 / APC-Alexa Fluor 750 (LS198-4-3, Beckman Coulter); CD20 / Pacific Blue (B9E9, Beckman Coulter), and CD45 / Krome Orange (J33, Beckman Coulter). During sample preparation a 10 µl of TdT / FITC (HT-6, Dako, Agilent (Santa Clara, CA, USA) and IgM / PE (Dako, Agilent) were added for intracellular staining Supp. Fig. 2. Permeabilization was performed using IntraPrep Permeabilization Reagent kit (Beckman Coulter) while following the manufacturer's instructions. Data were acquired on DxFLEX (Beckman Coulter) equipped with 405 nm, 488 nm, and 635 nm lasers. Data were analyzed using the Kaluza Analysis software version 2.1 (Beckman Coulter) following the gating strategy implemented based on the available literature (Suppl. Fig. 3). The target number of acquired events was 1,000,000 with minimum of 100,000 events to perform the analysis.

B cell lineage BCMA surface expression profiling and longitudinal B cell PB monitoring were assessed using two different spectral cytometry panels for a 5-laser Cytek Aurora (Cytek Biosciences, Fremont, CA, USA). Fresh PB and BM samples were processed following standard procedures and EuroFlow SOP. SpectroFlo QC beads (Cytek Biosciences) were used for routine instrument performance monitoring and automatic detector gain adjustment. Data were acquired using Cytek Aurora equipped with 355 nm, 405 nm, 488 nm, 561 nm, and 640 nm lasers. Spectral unmixing was performed using single-stain controls acquired from leukocytes or beads stained with the corresponding antibody–fluorophore conjugate, or with CD4 conjugated to the specific fluorophore, following best practices. Unmixing inaccuracies were examined in NxN plots and compensation was manually corrected when necessary by experienced data analyst. Isotype and FMO controls were used as negative control to determine the baseline for gating the frequency of BCMA+ events for each subset.

BCMA expression on individual B cell lineage subsets (pro-B, large pre-B, small pre-B, immature B cells, mature B cells, plasma cells) was evaluated with 12-colour spectral cytometry panel using the following reagents: CD38 / BUV496 (HIT2, BD), CD20 / PB (2H7, BioLegend), CD45 / PO (HI30, Invitrogen), IgM / BV650 (MHM-88, BioLegend), CD22 / BV786 (HIB22, BD), TdT / FITC (-, BQ), CD34 / PCP-Cy5.5 (8G12, BD), CD19 / RB780 (SJ25C1), BCMA and ISOTYPE / PE (19F2 / MOPC-173, BioLegend), CD39 / PE-Fire810 (A1, BioLegend), GPRC5D / ISOTYPE / APC (FAB6300A / 571961, R&D), and CD10 / APCH7 (HI10a, BD). Precursor subsets were gated in accordance with gating strategy utilized in conventional cytometry panel (Supp. Fig. 3). Plasma cells were gated as CD38high BCMA+ CD34- CD10-. Data were processed in Kaluza version 2.1 software (Beckman Coulter).

Longitudinal B cell lineage monitoring in PB was performed using a high-dimensional spectral cytometry panel developed and optimized at our center, and published as part of the Optimized Multicolor Immunophenotyping Panel (OMIP) collection (Venglar *et al*, 2025). The panel was optimized on numerous PB, BM, and frozen mononuclear cell samples and controls, with the overall optimization process spanning approximately 1.5 year, including several panel modifications. Reagents with the following specificity were used for surface staining: CD3, CD4, CD8, CD10, CD16, CD19, CD25, CD27, CD28, CD33, CD34, CD38, CD39, CD45, CD45RA, CD56, CD57, CD69, CD123, CD127, CD138, CD197, CD244 (2B4), CD272 (BTLA), CD226 (DNAM-1), igD, igG, igM, CD158b (KIR2DL), CD159a (NKG2A), CD159c (NKG2C), CD314 (NKG2D), CD335 (NKp46), CD279 (PD-1), TCRγδ, TIGIT, CD366 (TIM-3), and live/dead fixable blue. The PE channel is dedicated to CAR T cell detection using specific conjugates; however, it remains available for modification according to user preferences and experimental needs. Information on specific conjugates and clones, along with a full description of panel optimization and additional details, is available in the cited publication. Data were analyzed using the OMIQ platform (www.omiq.ai), incorporating conventional gating with standard cleaning of debris (SSC-A/FSC-A) and doublets (FSC-A, FSC-H), followed by gating of leukocytes (CD45+) and lymphoid cells (CD33-). Total B cells were gated as CD19+ CD3- CD56-, followed by gating of CD10low CD34- immature B cells and CD10- CD34- mature B cells. Mature B cells were divided into naive (CD27-) and memory (CD27+) subsets, in accordance with previously defined subsets by conventional cytometry, although the panel enables detailed characterization of mature B cells based on CD27,IgD, IgM, and IgG.

Single-cell RNA sequencing of NDMM patients and HD

BM aspirates were collected from 11 patients. B lineage cells were co-isolated by FACS according to a targeted panel including CD38, CD45, CD19 and CD138 for subsequent single-cell RNA and BCR sequencing (scRNA/BCR-seq). scRNA/BCR-seq was performed using 10X Genomics Single Cell 5′ Solution v1 and v2 kits following the manufacturer's protocol (10X Genomics, CA, USA). Libraries were sequenced in a NextSeq 550 (Illumina, CA, USA) and a HiSeq X (Illumina) for gene expression and BCR

data. Demultiplexing, alignment and different kit aggregation/correction, as well as gene counts and BCR reconstruction were performed with the Cell Ranger Software Suite (version 7.0, 10x Genomics), using default parameters and the GRCh38 reference genome. For validation, we used publicly available dataset of BM scRNA seq data https://explore.data.humancellatlas.org/projects/cc95ff89-2e68-4a08-a234-480eca21ce79) of 8 healthy donors (HD).

Analysis of single cell RNA sequencing data

Single-cell RNA/BCR-seq data was analyzed using the Python v3.11.6 and R v4.3.2 packages in accordance with best practices described previously (Heumos *et al*, 2023). Briefly, after Cell Ranger (v7.0, 10X Genomics; for demultiplexing, alignment and counts estimation), we performed quality control and filtering of all samples using mainly Scanpy toolkit (Wolf *et al*, 2018) and automatic thresholding and filtering based on median absolute divergence of the basic parameters; SoupX for correction of ambient RNA (Young & Behjati, 2020) and scDblFinder (Germain *et al*, 2022) for doublet identification and removal. Samples were then integrated into a single *anndata* object(Virshup *et al*, 2024) using Harmony (Korsunsky *et al*, 2019) for batch correction, followed by dimensionality reduction with UMAP.

Cell type annotation combined Leiden clustering, automated annotation with CellTypist (Immune_All_Low dataset(Domínguez Conde *et al*, 2022); Suppl. Fig. 12), and comparison with clusters from HDs reference data from the Single-cell Immune Cell Atlas of the Human Hematopoietic System (Broad Institute, Boston, USA; Suppl. Material). An ambiguous cluster between Small Pre-B and naïve B cells, annotated partially as Transitional B cells (by automated annotation), was classified as Immature B cells. Discrimination of tumor and normal PCs was performed using B-cell receptor sequencing (BCR) data on the Seurat toolkit (Hao *et al*, 2021) (Suppl. Methods). Publicly available HD data was preprocessed and integrated prior downloading. Therefore, we used only the annotation steps described above. All plots were prepared using Scanpy package.

Single-cell RNA comparison of clonal and normal PCs

The coparison of normal and clonal PCs was performed using R (version 4.1.2) with Seurat package (version 4.2.0) (Stuart *et al*, 2019). Matrices with filtered unique molecular identifier (UMI) counts from all cases were merged in a single Seurat object, and the BCR dataset was added. Cells without complete paired transcriptome and BCR information were excluded and additional filtering was performed using the following criteria: more than 3,000 expressed genes for the exclusion of doublets, less than 200

expressed genes, and/or a percentage of mitochondrial genes ≥10%. Normalization was performed using the global-scaling normalization function LogNormalize. Cluster detection was based on nearest-neighbor graphing (annoy method) and the Louvain algorithm, with a resolution of 1.5. Clusters were annotated manually based on expression of key lineage markers. Once annotated, cells from all patients and cohorts were integrated through reciprocal principal component analysis (rPCA) according to Seurat guidelines. Clonotypic BCR Ig gene rearrangements were considered when presenting a complete structure (i.e., both full IgH and IgL and detectable in ≥1 % of cells.

Supplementary Figures

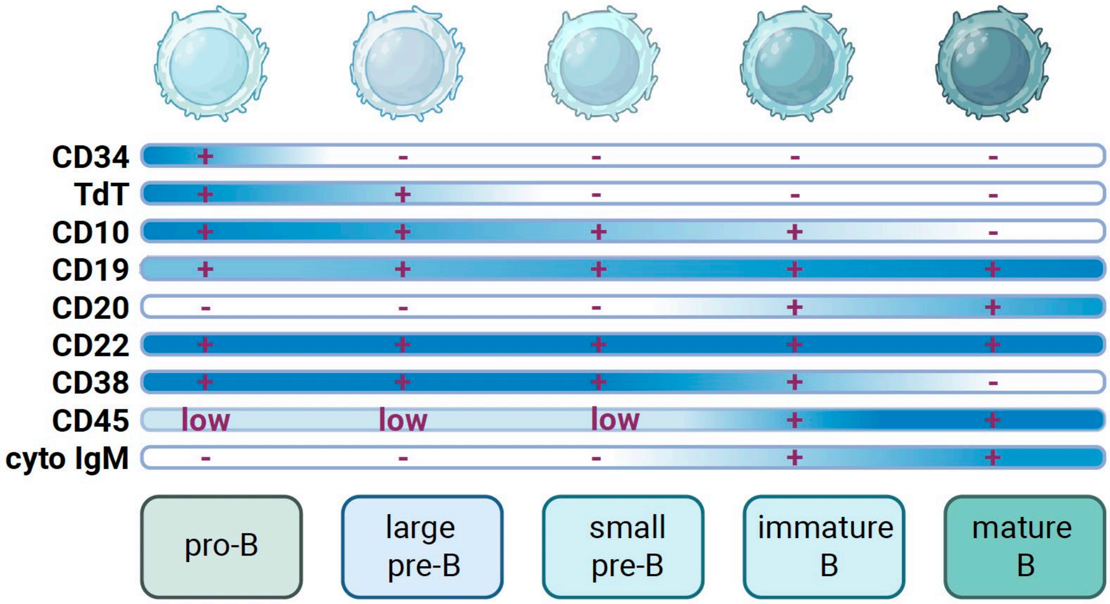

Supplementary Figure 1: Immunophenotypic description of B cell subsets. Expression patterns of key markers which were used to delineate individual stages from pro-B cells to mature B cells.

FITC	PE	PE-Daz 594	PerCP5.5	PC7	APC	APC-H7	PB	PO
cy TdT	cy IgM	CD22	CD34	CD19	CD10	CD38	CD20	CD45

Supplementary Figure 2: Flow cytometry panel for B cell precursors. A 9-colour conventional flow cytometry panel was developed to distinguish individual precursor stages (pro-B, large and small pre-B, and immature B cells), along with mature B cells and plasma cells. Intracellular markers are indicated in red.

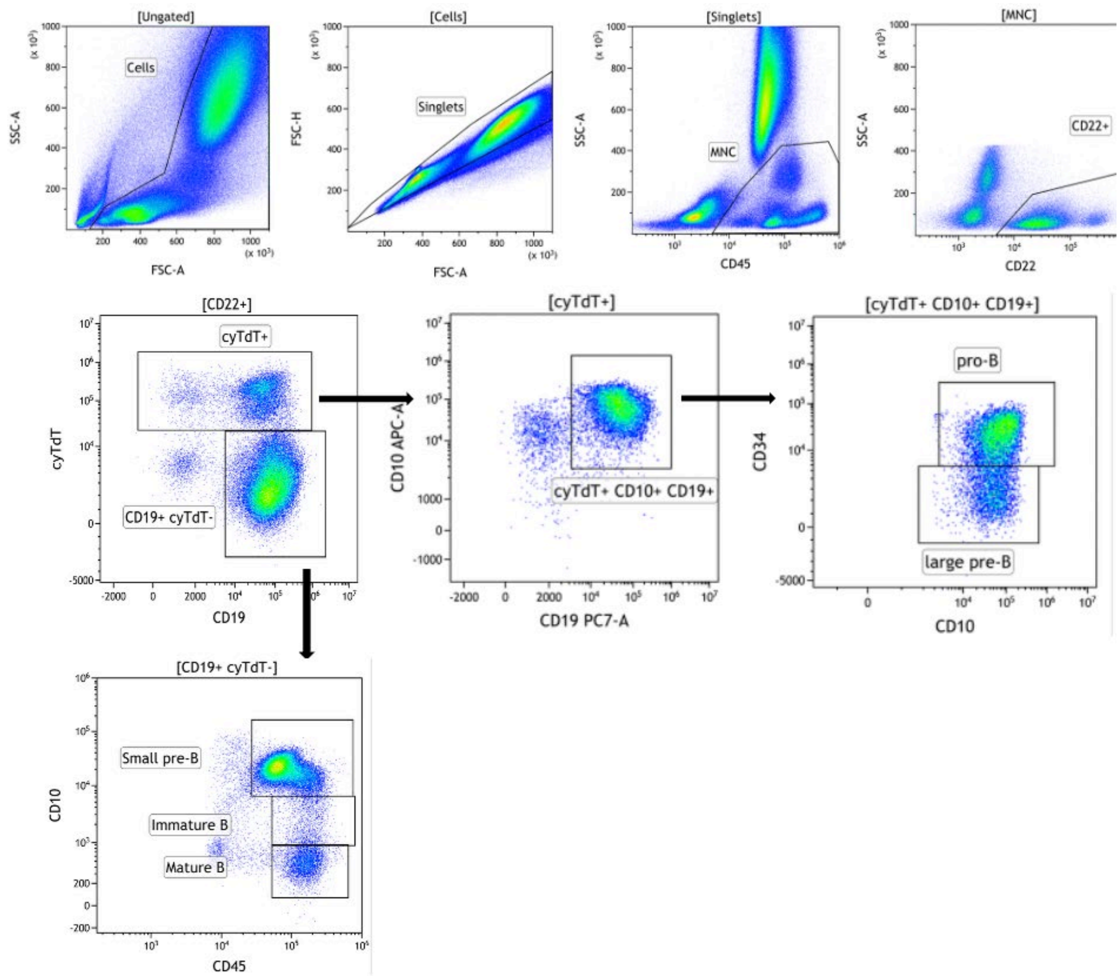

Supplementary Figure 3: Gating strategy for B cell precursors. Cells were initially cleaned to remove debris and doublets using light scatter parameters. Mononuclear cells (MNC) were then gated, followed by a selection of CD22+ SSClow cells, indicating B cell lineage commitment. The CD22+ cells were further divided into cyTdT+ CD19+/− (representing more immature cells) and cyTdT− CD19+ fractions. Within the cyTdT+ pool, the following precursor stages were identified: pro-B cells (CD19− CD10+ CD34+ CD45−) and large pre-B cells (CD19+ CD10+ CD34− CD45-). Within the cyTdT− CD19+ pool, the following stages were detected: small pre-B cells (CD10+ CD20− CD34− CD45low), immature B cells (CD10low CD20low CD34− CD45+), and mature B cells (CD19+ CD10− CD20+ CD34− CD45+).

BUV496	PB	PO	BV650	BV786	FITC	PerCPP5.5	RB780	PE	PE-Fire810	APC	APC-H7
CD38	CD20	CD45	IgM	CD22	cyTdT	CD34	CD19	BCMA	CD39	GPRC5D	CD10

Supplementary Figure 4: 12-Colour spectral flow cytometry panel for BCMA expression evaluation in B cell lineage subsets. The conventional B cell precursor panel was modified and expanded into a 12-colour full-spectrum cytometry panel to assess BCMA expression across various B cell subsets, including precursor stages (pro-B, large and small pre-B, and immature B cells), as well as mature B cells and plasma cells. Intracellular markers are indicated in red.

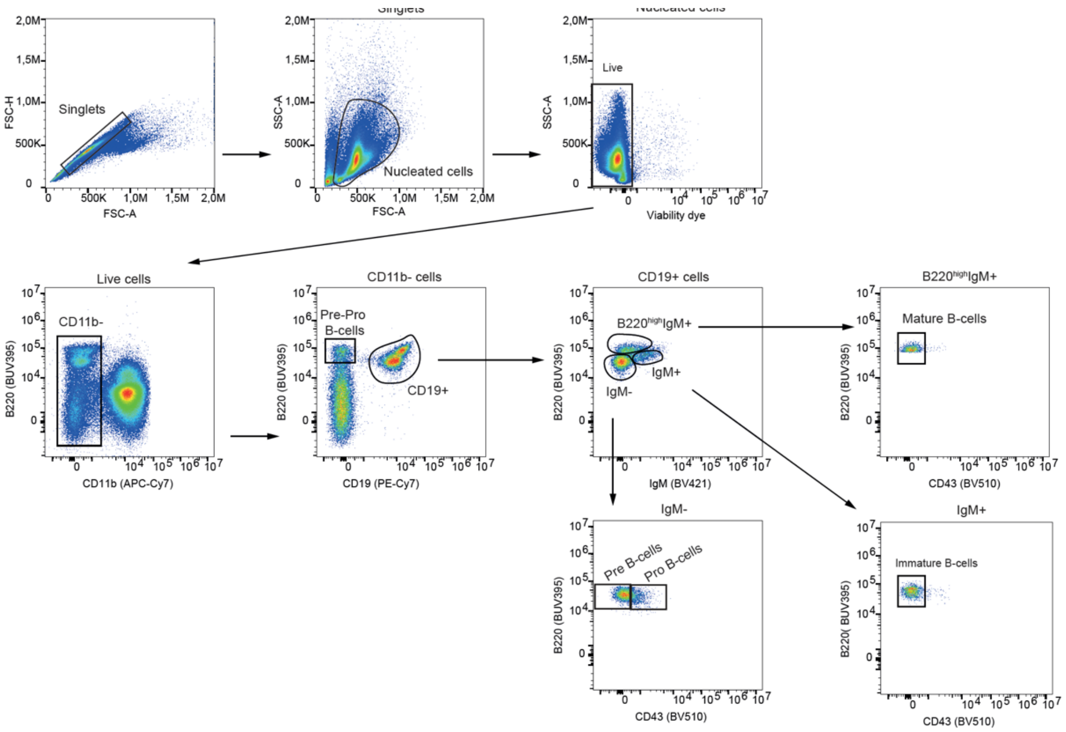

Supplementary Figure 5: Gating strategy for quantification of mouse B cell precursors and mature B cell subpopulations in the BM of MIcγ1 mice by multiparametric flow cytometry.

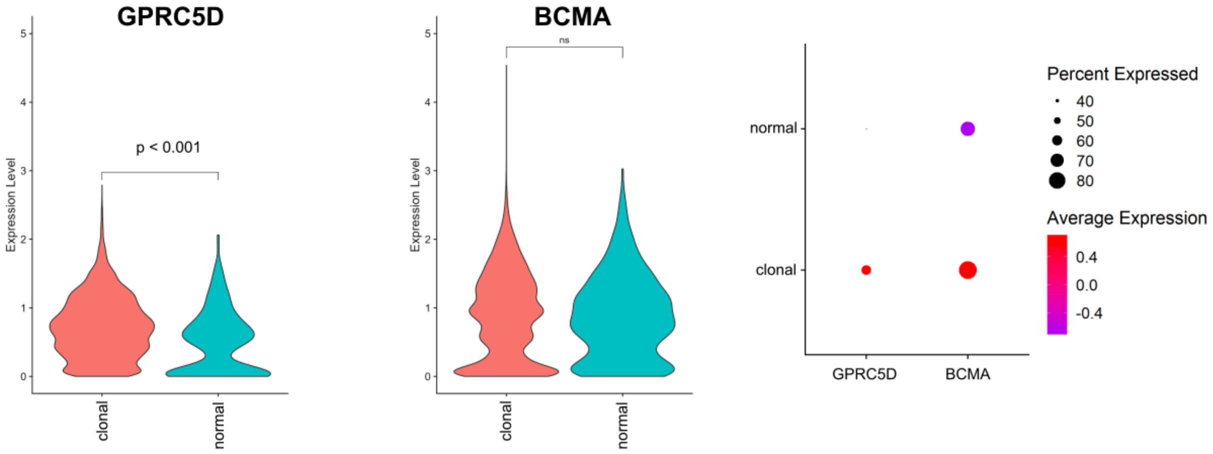

Supplementary Figure 6: scRNA-seq analysis comparing BCMA and GPRC5D expression in clonal versus normal plasma cells (PCs) from NDMM patients (N=11). Violin plots (left) illustrate significantly lower GPRC5D expression on normal PCs compared to clonal PCs, whereas there is no significant difference in BCMA expression between clonal and normal PCs (middle). Corresponding dot plots (right) further visualize these expression patterns, with dot size indicating the proportion of expressing cells and colour intensity representing the average gene expression level.

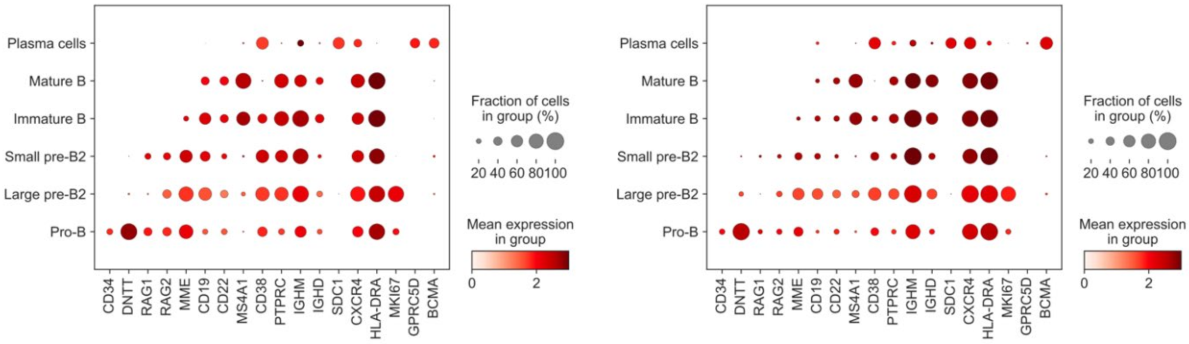

Supplementary Figure 7: Dot plot representation of marker gene expression utilized for manual curation of identified clusters in the multiple myeloma (MM; left) and healthy donor (HD; right) datasets. Dot size indicates the proportion of cells expressing each marker, and colour intensity represents the average expression level.

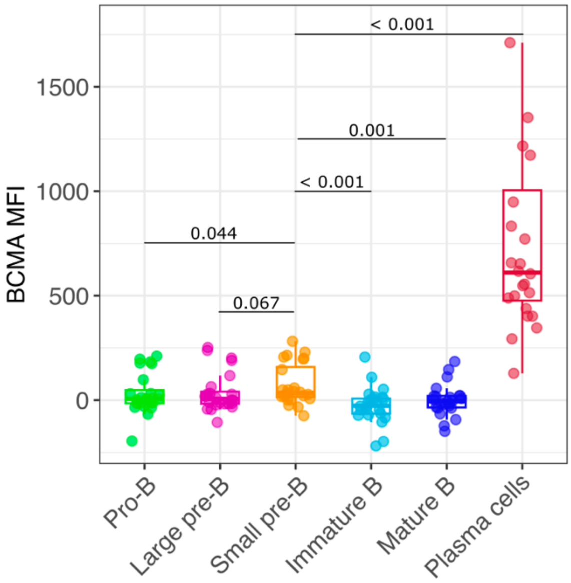

Supplementary Figures 8: Boxplots showing the median fluorescence intensity (MFI) of BCMA (Biolegend, PE, clone 19F2) across different stages of B-cell development and on plasma cells, assessed by spectral flow cytometry in bone marrow samples from 24 NDMM patients.

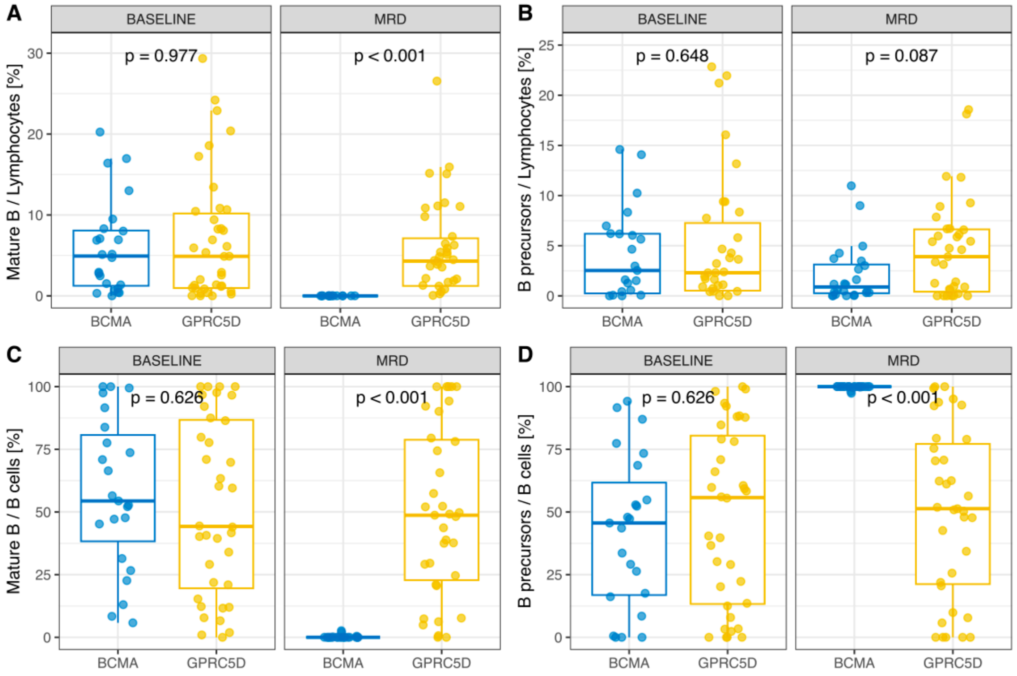

Supplementary Figure 9: Comparison of mature B cells and B cell precursors in BCMA versus GPRC5D-directed therapy groups (N=62, paired samples). Boxplots display frequencies of mature B cells and B cell precursors at baseline and MRD timepoints in bone marrow samples from patients treated with BCMA-targeted (blue) or GPRC5D-targeted (yellow) treatment. (A) Proportions of mature B cells normalized to total lymphocytes. (B) Proportions of B cell precursors normalized to total lymphocytes. (C) Proportion of mature B cells within total B cells. (D) Proportion of B cell precursors within total B cells. Statistical comparisons between groups were performed using Wilcoxon rank-sum test, with p-values indicated above each comparison. Significant reductions in mature B cell proportions were observed after anti-BCMA treatment (A, C), while the GPRC5D group retained higher mature B cell presence. No significant difference was observed in the level of B precursors after anti-BCMA vs anti-GPRC5D treatments (B).

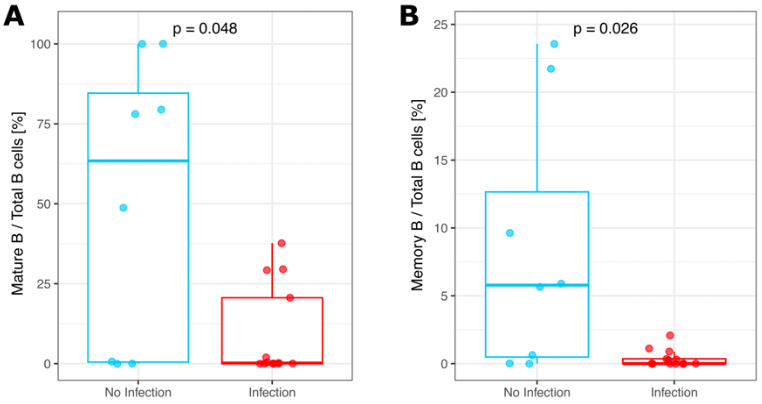

Supplementary Figure 10: Reduced levels of mature B **(A)** and memory B **(B)** cells among total B cells in patients with infection following treatment with anti-BCMA or anti-GPRC5D monotherapy (N=28).

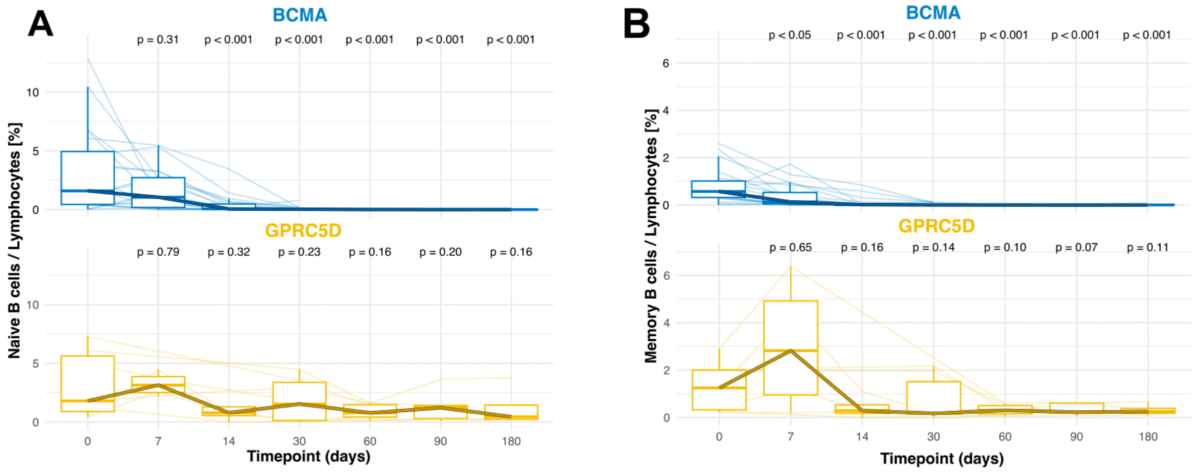

Supplementary Figure 11: Longitudinal monitoring of the effects of bsAbs targeting BCMA and GPRC5D in PB of 27 MM patient (BCMA: N=20, GPRC5D: N=7). In total, 161 measurements were performed (BCMA: N=122; GPRC5D: N=39) at following timepoints: at baseline and on days 7, 14, 30, 60, 90, and 180 on **(A)** naïve B cells and **(B)** memory B cells, assessed using a OMIP panel by spectral cytometry.

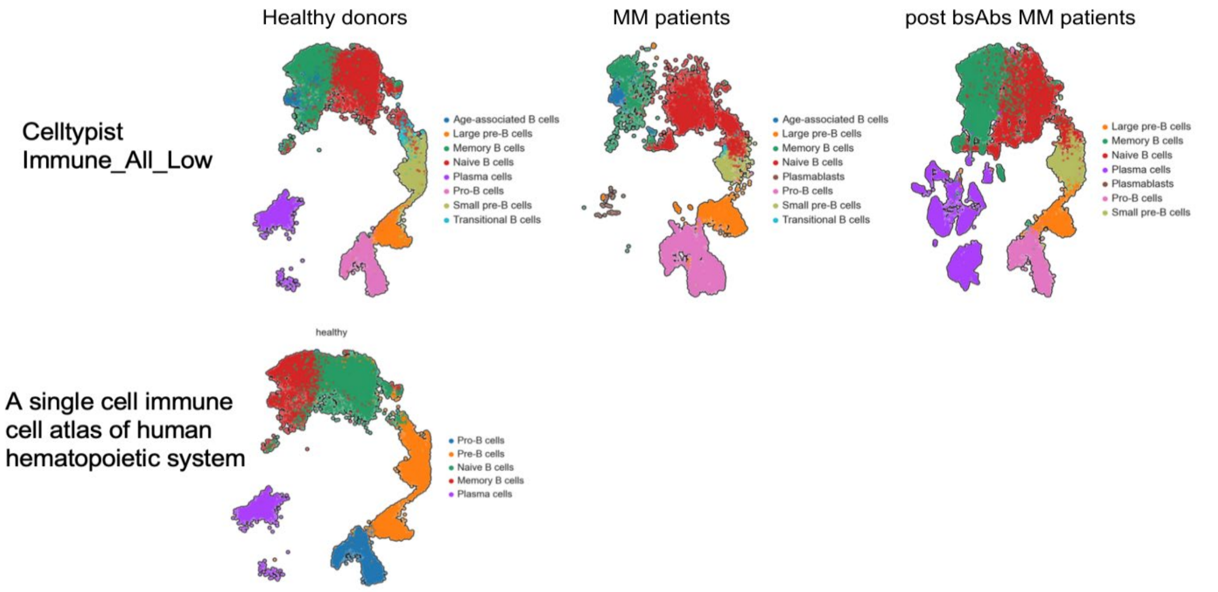

Supplementary Figure 12: Automated annotation of single cell data by Celltypist Immune_All_Low atlas (top) and annotation from A single cell immune atlas of human hematopoetic system (bottom).

Supplementary References

Domínguez Conde, C., Xu, C., Jarvis, L.B., Rainbow, D.B., Wells, S.B., Gomes, T., Howlett, S.K., Suchanek, O., Polanski, K., King, H.W., Mamanova, L., Huang, N., Szabo, P.A., Richardson, L., Bolt, L., Fasouli, E.S., Mahbubani, K.T., Prete, M., Tuck, L., Richoz, N., et al (2022) Cross-tissue immune cell analysis reveals tissue-specific features in humans. *Science (New York, N.Y.)*, **376,** eabl5197.

Germain, P.-L., Lun, A., Meixide, C.G., Macnair, W. & Robinson, M.D. (2022) Doublet identification in single-cell sequencing data using *scDblFinder*. Available at: https://f1000research.com/articles/10-979 [Accessed January 10, 2025].

Hao, Y., Hao, S., Andersen-Nissen, E., Mauck, W.M., Zheng, S., Butler, A., Lee, M.J., Wilk, A.J., Darby, C., Zager, M., Hoffman, P., Stoeckius, M., Papalexi, E., Mimitou, E.P., Jain, J., Srivastava, A., Stuart, T., Fleming, L.M., Yeung, B., Rogers, A.J., et al (2021) Integrated analysis of multimodal single-cell data. *Cell*, **184,** 3573-3587.e29.

Heumos, L., Schaar, A.C., Lance, C., Litinetskaya, A., Drost, F., Zappia, L., Lücken, M.D., Strobl, D.C., Henao, J., Curion, F., Schiller, H.B. & Theis, F.J. (2023) Best practices for single-cell analysis across modalities. *Nature Reviews Genetics*, **24,** 550–572.

Korsunsky, I., Millard, N., Fan, J., Slowikowski, K., Zhang, F., Wei, K., Baglaenko, Y., Brenner, M., Loh, P. & Raychaudhuri, S. (2019) Fast, sensitive and accurate integration of single-cell data with Harmony. *Nature Methods*, **16,** 1289–1296.

Orfao, A., Matarraz, S., Pérez-Andrés, M., Almeida, J., Teodosio, C., Berkowska, M.A. & van Dongen, J.J.M. (2019) Immunophenotypic dissection of normal hematopoiesis. *Journal of Immunological Methods*, **475,** 112684.

Stuart, T., Butler, A., Hoffman, P., Hafemeister, C., Papalexi, E., Mauck, W.M., Hao, Y., Stoeckius, M., Smibert, P. & Satija, R. (2019) Comprehensive Integration of Single-Cell Data. *Cell*, **177,** 1888-1902.e21.

Venglar, O., Radova, E., Broskevicova, L., Hajek, R. & Jelinek, T. (2025) 40-Parameter/37-Color Spectral Cytometry Panel for Robust Immunoprofiling of Human Lymphoid Subsets in Cancer Patients. *Cytometry. Part A: The Journal of the International Society for Analytical Cytology*.

Virshup, I., Rybakov, S., Theis, F.J., Angerer, P. & Wolf, F.A. (2024) anndata: Access and store annotated data matrices. *Journal of Open Source Software*, **9,** 4371.

Wolf, F.A., Angerer, P. & Theis, F.J. (2018) SCANPY: large-scale single-cell gene expression data analysis. *Genome Biology*, **19,** 15.

Young, M.D. & Behjati, S. (2020) SoupX removes ambient RNA contamination from droplet-based single-cell RNA sequencing data. *GigaScience*, **9,** giaa151.
